# Epigenetic Analyses of Alcohol Consumption in Combustible and Non-Combustible Nicotine Product Users

**DOI:** 10.3390/epigenomes5030018

**Published:** 2021-09-01

**Authors:** Kelsey Dawes, Luke Sampson, Rachel Reimer, Shelly Miller, Robert Philibert, Allan Andersen

**Affiliations:** 1 Department of Psychiatry, University of Iowa, Iowa City, IA 52242, USA; luke-sampson@uiowa.edu (L.S.); Robert-philibert@uiowa.edu (R.P.); allan-andersen@uiowa.edu (A.A.); 2 Behavioral Diagnostics LLC, Coralville, IA 52241, USA; smiller@bdmethylation.com; 3 College of Public Health, Des Moines University, Des Moines, IA 50312, USA; rachel.reimer@dmu.edu

**Keywords:** epigenetics, DNA methylation, alcohol use disorder, nicotine, smoking

## Abstract

Alcohol and tobacco use are highly comorbid and exacerbate the associated morbidity and mortality of either substance alone. However, the relationship of alcohol consumption to the various forms of nicotine-containing products is not well understood. To improve this understanding, we examined the relationship of alcohol consumption to nicotine product use using self-report, cotinine, and two epigenetic biomarkers specific for smoking (cg05575921) and drinking (Alcohol T Scores (ATS)) in *n* = 424 subjects. Cigarette users had significantly higher ATS values than the other groups (*p* < 2.2 × 10^−16^). Using the objective biomarkers, the intensity of nicotine and alcohol consumption was correlated in both the cigarette and smokeless users (*R* = −0.66, *p* = 3.1 × 10^−14^; *R*^2^ = 0.61, *p* = 1.97 × 10^−4^). Building upon this idea, we used the objective nicotine biomarkers and age to build and test a Balanced Random Forest classification model for heavy alcohol consumption (ATS > 2.35). The model performed well with an AUC of 0.962, 89.3% sensitivity, and 85% specificity. We conclude that those who use non-combustible nicotine products drink significantly less than smokers, and cigarette and smokeless users drink more with heavier nicotine use. These findings further highlight the lack of informativeness of self-reported alcohol consumption and suggest given the public and private health burden of alcoholism, further research into whether using non-combustible nicotine products as a mode of treatment for dual users should be considered.

## 1. Introduction

Tobacco and alcohol use is independently associated with high rates of morbidity and mortality worldwide, with concurrent use potentiating the health risks of either substance alone [1,2,3,4,5]. The two substances are highly comorbid, with estimates up to 92% of individuals in the U.S meet the DSM-V diagnostic criteria for alcohol use disorder (AUD) report smoking cigarettes [6,7]. Similarly, cigarette users have a 95% greater risk of alcohol dependency and report higher levels of chronic alcohol use and binge drinking compared to non-smokers [8,9].

Prior studies suggest the high prevalence of alcohol and tobacco concurrent use could be the result of mutual psychosocial factors and two neurobiological mechanisms: cross-reinforcement and cross-tolerance [10,11,12,13,14,15,16]. Cross-reinforcement refers to the ability of alcohol and tobacco to enhance the motivation to consume the other via activation of the mesolimbic dopamine pathway. Cross-tolerance occurs when the repeated use of both alcohol and tobacco results in the reduction of response to one substance by the use of the other, subsequently leading to an escalation of substance use to achieve the same response. Through these interactive effects, the use of one substance increases the amount consumed of the other leading to the progression of alcohol and tobacco-associated pathology.

Critically, concurrent use of alcohol and tobacco pose significant barriers to cessation efforts, with the use of one substance hindering the cessation attempts of the other. Alcohol withdrawal symptoms have been reported to be more severe in individuals who smoke, with lower rates of long-term alcohol abstinence [17,18]. Similarly, current or past AUD decreases the likelihood of smoking cessation [19,20]. Although a variety of evidence-based pharmacological and behavioral treatments for tobacco and alcohol use exist, the best treatment strategy for those with comorbid additions remains unclear. Specifically, clinicians are uncertain if they should treat both additions together or separately. In order to most effectively address the total burden of illness, clinicians engaged in alcohol or smoking cessation treatment should screen for comorbid use, and consider alcohol and tobacco polysubstance abuse as a singular condition. 

The development and implementation of effective treatment strategies for nicotine and alcohol polysubstance abuse are impeded by the shortcomings in our current understanding of the relationship between what is now covered under the term “tobacco use disorder” and AUD. Prior to this past decade, studies of “tobacco use disorder” focused on cigarette users, as cigarettes were the major form of tobacco use in industrialized countries. However, recent advancements in nicotine delivery systems have since altered the epidemiology and nosology of the use of tobacco and tobacco-related products [21]. Since their first appearance in 2006, the prevalence of electronic nicotine delivery devices (ENDS; a term including e-cigarettes and vape pens) grew rapidly accompanied by a proportional decrease in cigarette use [22]. The DSM V specifies the use of tobacco in the “A” criteria for the diagnosis of Tobacco Use Disorder and does not directly address the pure use of nicotine in the main portion of the description of Tobacco Use Disorders (page 571–577) [21,23]. In response to this, some investigators have introduced the term “Nicotine Use Disorder” as a more embracing categorization [24,25,26]. However, Nicotine Use Disorder is not specifically mentioned in the DSM V. Furthermore, it is not clear if the morbidity and mortality associated with non-combustible nicotine use are similar to that of smoking [27]. Determining the morbidity and mortality associated with ENDS use is of extreme interest to clinicians, researchers, policymakers, and actuaries. 

A second hindrance to understanding nicotine and alcohol polysubstance use is the inability to reliably detect nicotine and problematic alcohol consumption. The default method relies upon the patient’s self-report, which has been shown in several studies to be unreliable [28,29,30]. Similarly, the self-report of ENDS use may not be accurate. Following up on concerns raised by Rubinstein and associates [31], Goniewicz and colleagues examined urine samples from Population Assessment of Tobacco and Health (PATH) study subjects who self-reported exclusive ENDS use [32]. They found that 15% of the self-reported exclusive ENDS users had significantly elevated levels of NNAL, a biomarker of exposure to tobacco-specific nitrosamine NNK, in their urine. Because Goniewicz only examined a single time point in each subject and NNAL is capable of detecting combustible tobacco use only over the past several days, this raises the possibility that many other self-reported exclusive ENDS users in the PATH study may have also been smoking outside of the one-month time window quantified by NNAL [33]. If so, a substantial number of self-reported exclusive ENDS users may have histories of combustible tobacco use whose effects could significantly affect conclusions from the study.

Biological biomarkers have been developed in attempts to overcome the challenges of unreliable self-report. Algorithms incorporating several different liver enzymes and ethanol metabolites are frequently used to ascertain an individual’s self-report of alcohol use, as well as the frequency and duration of use [34]. As a number of hepatological conditions produce elevated levels of liver enzymes, these biomarkers have low specificity. While ethanol metabolites are relatively unaffected by underlying pathological conditions, their detection time window of <90 h limits their clinical utility. Two biomarkers are currently used in the clinic to detect smoking and/or nicotine use: exhaled carbon dioxide (CO) and cotinine [35]. Whereas CO measurements are easy and inexpensive to perform, its ability to detect smoking only within the past 3–4 h renders it insensitive to episodic or light smoking patterns [35,36]. Furthermore, given the frequent comorbid patterns of substance use, the potential false positives from combustible marijuana use further hinder its utility [37]. Cotinine is a metabolite of nicotine with a detection time window of approximately 48 h after the last use of a nicotine-containing product. While cotinine assessments are more sensitive than CO [38], the inability of cotinine to distinguish the source of nicotine exposure renders it uninformative in light of modern nicotine delivering devices. The ability of a biomarker to distinguish the source of nicotine exposure is imperative for several reasons: (1) There are differences in the associated health risks due to the use of cigarettes, ENDS, and smokeless tobacco, (2) Patients often use nicotine replacement therapy (NRTs) in smoking cessation efforts to reduce physical withdrawal symptoms. Potential false positives of cotinine assessments obstruct the ability of a clinician to adequately monitor treatment response and maintaining abstinence.

The incorporation of precision epigenetic assays targeting highly predictive loci for both smoking and alcohol consumption into new studies of non-combustible forms of nicotine may address some of the shortcomings for understanding alcohol and nicotine polysubstance use. Building on genome-wide DNA methylation analyses, we have developed a set of methylation-sensitive digital PCR (MSdPCR) assays for predicting alcohol and cigarette consumption that can be measured in whole blood or saliva [39,40,41]. To date, cg05575921 is the strongest and most consistently reported CpG site associated with smoking [42]. This residue is located within the aryl hydrocarbon receptor repressor (AHRR) gene, a known mediator for the detoxification of the polyaromatic hydrocarbons (PAH) produced from cigarette consumption [43]. The smoking-induced methylation alterations of cg05575921 are specific to combustible nicotine products and are not confounded by other sources of nicotine [44]. We have demonstrated that MSdPCR assessments of whole blood cg05575921 methylation can be used to accurately determine smoking status in adults with an ROC of 0.99 and an inter-assay variation of 0.7% [41]. These findings have been extended to adolescents with light-smoking and episodic behavioral use patterns [40]. Using this assay, we have refined the dose-dependent demethylation response in adults, with a decrease of 1% being equivalent to the consumption of 1.2 cigarettes per day. An intensity-dependent reversion of cg05575921 methylation is seen as a function of smoking cessation, accentuating the clinical utility for monitoring cessation therapy [45].

The MSdPCR panel for alcohol is more recently described. We devised an Alcohol T Score metric calculated from the methylation values of four loci, cg02583484, cg04987734, cg09935388, and cg04583842 [39]. We have demonstrated that the ATS outperforms the CDT with an AUC of 0.96 for detecting those with heavy alcohol consumption [46]. Similar to cg05575921, one of the alcohol-sensitive loci demonstrated reversion to baseline methylation levels with treatment enforced alcohol abstinence [39].

In this communication, we use objective epigenetic biomarkers to assess the co-morbid relationship of alcohol and nicotine consumption in a biochemically verified cohort of exclusive cigarette, ENDS, smokeless tobacco, and non-nicotine users.

## 2. Results

The key demographic and clinical characteristics of the 424 subjects are given in Table 1. The vast majority of subjects in each group were White (82–95%), with the smokeless users being almost exclusively White (95%). With the exception of the smokeless users, subjects were predominately female (5% vs. 61–69%, respectively). The ENDS users were significantly younger than the other groups, with an average age of 22.7 (*t* = 9.90, *p* = 4.903 × 10^−16^). The cigarette and smokeless users had an average age of 40.9 and 36.1, respectively. Both groups were significantly older than the controls (*t* = 7.92, *p* = 2.54 × 10^−13^ and *t* = −2.23, *p* = 0.0319, respectively), but did not differ from each other.

With respect to self-reported substance-use variables, a total of 122 and 21 subjects reported a history of nicotine and alcohol dependence respectively, with there being no significant differences between the three nicotine use groups. Notably, 95% of those who reported alcohol dependence also reported nicotine dependence. The ATS was significantly higher in those who reported both nicotine and alcohol dependence compared to the other groups combined (Supplemental Figure S1; *t* = −4.75, *p* = 1.14 × 10^−04^). Finally, 24 subjects reported drinking an average of 2 or more drinks per day with control subjects being less likely than nicotine users to report consuming 2 or more drinks per day (*p* < 0.0001) but there are no significant differences between the nicotine user groups.

**Figure 1:**
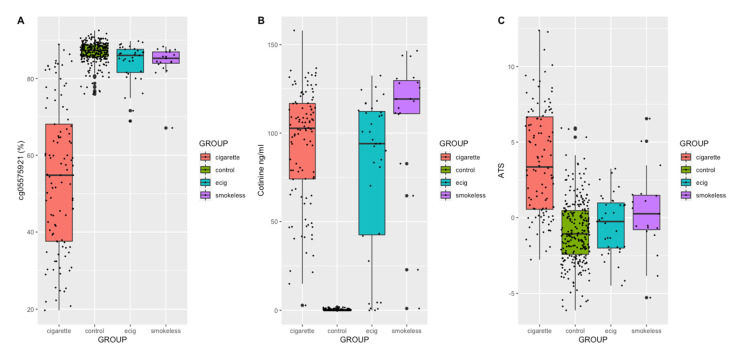
Distribution of cg05575921 methylation (**A**), cotinine (**B**), and ATS (**C**) values by user group.

Figure 1 illustrates the distribution of cg05575921 methylation (A), serological cotinine (B), and ATS (C) as a function of user status, while average values for each group are provided in Table 1. Whereas all nicotine use groups demonstrated markedly elevated serum cotinine values as compared to controls (*p* < 2 × 10^−16^), there was only a slight variability of cotinine between the nicotine user groups. Smokeless users had significantly higher cotinine levels than the ENDS users (*t* = −2.5, *p* = 0.016); however, no other significant inter-group differences were seen. As expected, the cigarette users had significantly lower cg05575921 methylation as compared to the other three groups (*p* < 2 × 10^−16^). There was also evidence of subtle but significant differences in methylation between controls and ENDS users (*t* = 2.95, *p* < 0.01) and between controls and smokeless users (*t* = 2.32, *p* < 0.05). A total of 4 non-cigarette users had cg05575921 methylation values < 75%, with 2 of these subjects having elevated exhaled CO levels.

We next examined the relationship of the subjective self-reported drinking frequency to the objective ATS value. ATS value significantly varied as a function of self-reported drinking frequency (Supplemental Figure S2, *p* < 0.001). Significant differences in the average ATS values were seen between those who reported no alcohol use those who reported either moderate (8 to 14 drinks per week) (−0.12 vs. 1.46; *t* = 2.55, *p* = 0.014) or heavy alcohol use (14+ drinks per week) (−0.12 vs. 2.35; *t* = 2.93, *p* = 0.007). A significant difference was also observed between those who reported light alcohol use (1 to 7 drinks per week) with those who reported moderate (0.19 vs. 1.46; *t* = −2.12, *p* = 0.04) and heavy alcohol use (0.19 vs. 2.35; *t* = −2.61, *p* = 0.015). Interestingly, there were no observed differences in the average ATS value between those of moderate to those with heavy alcohol use, and between those who reported no use to those who reported light alcohol use.

**Figure 2:**
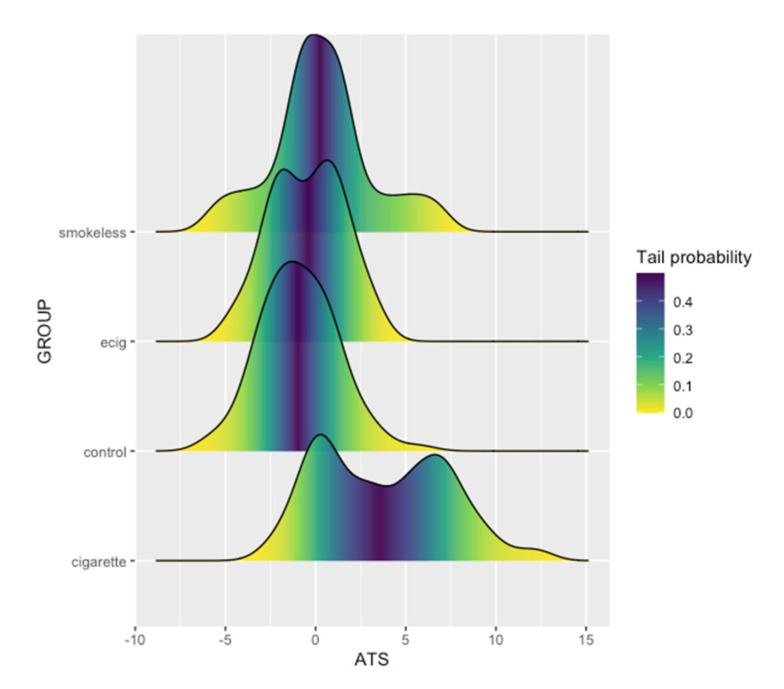
Density plot of the ATS distribution stratified by nicotine use group.

**Figure 3:**
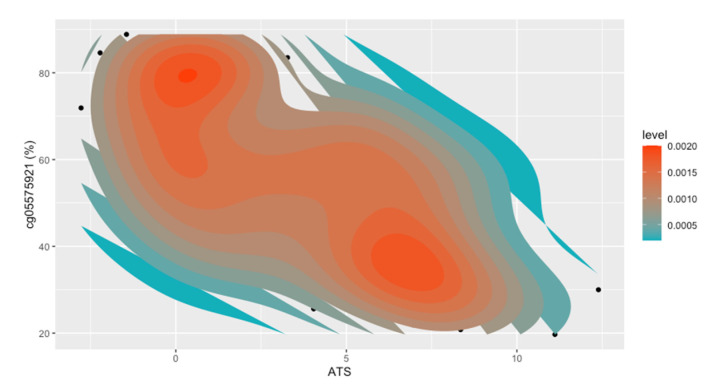
A 2D kernel density estimation plot illustrating the negative correlation (Pearson’s, *R* = −0.66, *p* = 3.14 × 10^−14^) between cg05575921 methylation and ATS in cigarette users.

To gain a better understanding of the comorbid relationship between alcohol consumption and different nicotine products, we next examined ATS levels as a function of user status. As illustrated in Figure 1C, cigarette users had significantly higher ATS levels than any of the other nicotine user groups (*t* = −8.31, *p* = 6.531 × 10^−14^). Apart from the cigarette users, we did not find significant differences in the ATS between the other user groups. The density plot of ATS in the non-cigarette users shows a unimodal distribution, with a slight dip in the ENDS users (Figure 2). In contrast, the density plot of the cigarette users shows a distinct bimodal distribution. To better understand the biological basis of this bimodal distribution, we further classified smokers into two groups based on the ATS node of each peak in the bimodal distribution. Cigarette users with ATS values indicative of light to moderate drinking, found in the left peak, were categorized as low alcohol-consuming smokers (LAS), with a maximum ATS value of 3.71 (*n* = 56). Cigarette users with ATS values indicative of moderate to heavy drinking, found in the right peak, were categorized as heavy alcohol-consuming smokers (HAS), with a minimum ATS of 3.72 (*n* = 52). There was a prominent difference in the average cg05575921 methylation values between the LAS and HAS (65% vs. 43%; *t* = −7.23, *p* = 8.78 × 10^−11^), despite only a small difference seen in the serum cotinine values (87 ng/mL vs. 100 ng/mL; *t* = 2.08, *p* = 0.04). To that end, we used a 2D kernel density estimation plot (Figure 3) to illustrate the positive correlation between smoking intensity and alcohol consumption (*R* = −0.66, *p* = 3.1 × 10^−14^), with the ATS value of the two clusters aligning with the average ATS found within each node of the bimodal distribution.

We next used linear regression to examine the relationship between the objective nicotine and alcohol biomarkers controlling for age (Supplemental Table S1). By itself, cotinine levels were significantly correlated to ATS only in the smokeless group (*R*^2^ = 0.6128, *p* = 1.97 × 10^−4^). In contrast, cg0557921 was significantly correlated across all nicotine consuming subjects and cigarette users (*R*^2^ = 0.6105, *p* < 2.2 × 10^−16^ and *R*^2^ = 0.5374, *p* < 2.2 × 10^−16^, respectively), while a trend for association was observed in ENDS users (*R*^2^ = 0.1203, *p* = 0.0554).

**Figure 4:**
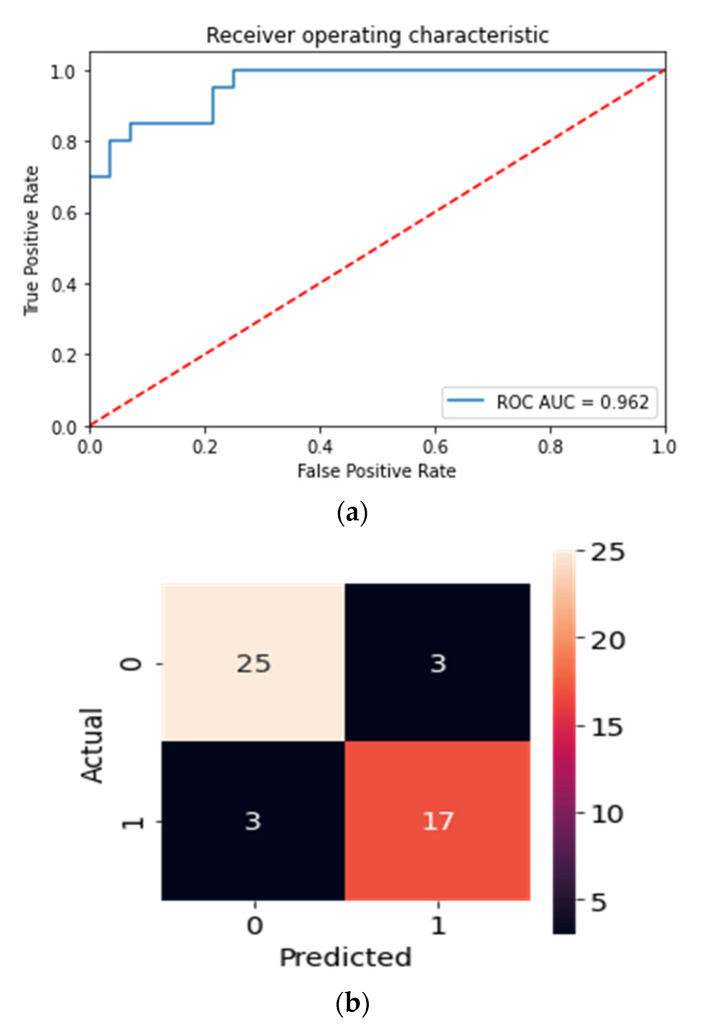
Receiver operator characteristic with the area under the curve value (**a**) and confusion matrix (**b**) of the Balanced Random Forest Classifier for HAC status in nicotine user groups using cg05575921, cotinine, and age as features.

Finally, to determine the ability of the objective nicotine biomarkers to predict heavy alcohol consumption (HAC) in those who consume nicotine, a classifier was built using cotinine, cg05575921, and age. Using a repeated stratified k-fold cross-validation approach, a Balanced Random Forest model with random under-sampling was built to predict HAC. HAC status was determined for each nicotine-consuming subject by binning the ATS value, with the criteria adapted from the average ATS value of those who self-reported a drinking frequency of at least 14 drinks per week within the past year. Hence, HAC was coded as a binary variable representing those with HAC (ATS > 2.35) and without HAC (ATS < 2.35). The key clinical and demographic characteristics of the HAC case and controls subjects are provided in Supplemental Table S2. The model performed well with a Receiver Operator Characteristic (ROC) Area Under the Curve (AUC) of 0.962, 87.5% accuracy, 89.3% sensitivity, and 85% specificity. The plots of the ROC curve and confusion matrix are provided in Figure 4a,b, respectively.

## 3. Discussion

In this study, we used objective DNA methylation biomarkers to assess the comorbid relationship of alcohol and nicotine consumption in a biochemically verified cohort of exclusive cigarette, ENDS, smokeless tobacco, and non-nicotine users. In summary, the current results demonstrate that smoking is associated with greater alcohol consumption than non-combustible nicotine use and nicotine abstention, and non-combustible nicotine users do not drink more than nicotine abstinent controls. Notably, our results also demonstrate that heavy cigarette and smokeless users consume more alcohol than light users. The findings also highlight the advantages of using objective biomarkers to unambiguously identify and quantify alcohol and cigarette consumption.

Although the sample size for non-combustible nicotine users is small, we believe the current study highlights the potential for Precision Epigenetic biomarkers to provide clinicians an additional tool for screening and monitoring alcohol and nicotine consumption. Additional limitations include subject ascertainment from a single Midwestern state, and our strict inclusion criteria requiring exclusive use of one form of nicotine or complete abstinence may have contributed to increased heterogeneity between groups.

The current findings will be particularly useful for those seeking a better understanding of non-combustible nicotine use and alcohol consumption. While the interactions between alcohol and nicotine are complex, the comorbid relationship between alcohol and cigarette use is well documented. Conversely, there is a limited body of literature assessing the dual-use and incurred morbidity associated with the concurrent use of non-combustible nicotine and alcohol. Our findings that smokeless tobacco users consumed less alcohol than cigarette users but more than non-smoking subjects are consistent with previous studies from Zhou and colleagues [47]. Comparatively, our finding that ENDS users do not consume significantly more alcohol than nicotine abstinent controls may be surprising given the prior literature. Recent studies have shown that ENDS users have an increased risk of alcohol misuse and alcohol use disorder when compared to non-users [48,49]. It is important to note that these studies relied on self-reported alcohol and nicotine use, and included dual (ENDS and cigarette) users. As our study focused only on adults, a parsimonious interpretation is that we were merely underpowered to detect a positive association of ENDS uses with alcohol consumption. A distinguished advantage of the cg05575921 assay is that it allows us to detect and quantify smoking, even in the face of confounding nicotine exposure. Hence, we were able to detect concealed smoking in our self-reported “exclusive” ENDS users. While there was a significant relationship between cg05575921 and ATS values, a relationship between ATS and cotinine was not seen. This suggests that dual cigarette and ENDS use, but not exclusive ENDS use, may be associated with increased alcohol consumption.

The concurrent alcohol and nicotine consumption patterns seen in cigarette and smokeless tobacco users were strikingly different than those observed in ENDS users. Whereas subjects who smoked consumed more alcohol than any other nicotine-use group, the use of a dichotomized smoking status alone would yield a low specificity for predicting heavy alcohol consumption due to the heterogeneous behavioral use patterns seen within smokers. Using our DNA methylation biomarkers, we demonstrated that light smokers consumed less alcohol than heavy smokers. While not as prominent, this heterogeneity was observed in smokeless tobacco users as well. Light smokeless tobacco users concomitantly drank less alcohol than heavy users, determined by cotinine and ATS measurements. The inter- and intra-nicotine differences of dual consumption patterns seen between the nicotine user groups illustrated in this report underscore the necessity for clinical biomarkers that are able to distinguish between the different sources of nicotine and quantify current and cumulative exposures of both alcohol and nicotine. To further emphasize the implications of these differences, we demonstrated that a model incorporating the objective nicotine biomarkers and age was able to predict heavy alcohol consumption with an ROC AUC of 0.962.

It is important to note that while many of the analyses using the epigenetic assessments of alcohol consumption were significant, there were no differences in the self-reported use of alcohol between the nicotine user groups. This may suggest that the ATS metric is more reliable than the self-report method of alcohol consumption. Whereas this statement may be true, the ATS metric was designed to detect heavy alcohol consumption. Its ability to differentiate amidst lower levels of consumption has never been established among well-calibrated drinkers. With that said, the ATS value was able to distinguish between those who reported no-to-light alcohol use and those who reported moderate-to-heavy use but not between no-to-light or moderate-to-heavy use. To that end, the correlation between the ATS and age may be indicative of the cumulative alcohol exposure over time that is required to manifest aberrations in DNA methylation at these specific loci. While more research is needed to refine the dose–response of the ATS value, these findings further support the clinical utility of the ATS as a biomarker for identifying those with problematic alcohol consumption that may benefit from additional testing and treatment.

In conclusion, we report the incorporation of objective MSdPCR assays for alcohol consumption and smoking could provide a powerful framework for studying the nature of concurrent alcohol and nicotine use. While the use of ENDS to promote smoking cessation remains controversial, our report raises the possibility of synergistic alcohol cessation efforts by way of concurrent cigarette users switching to smokeless forms of nicotine. Given the prevalence and significant morbidity associated with smoking and heavy alcohol use, a reduction in alcohol consumption in smokers who switch to ENDS could have profound public health implications. Further research is needed to better understand the risks and benefits of such a strategy for cigarette users.

## 4. Materials and Methods

Subjects: The subjects whose data are featured in this study were ascertained between July 2019 and March 2020. These participants were solicited through mass email to the University of Iowa and Des Moines University community, social media ads, radio ads, and fliers. Each form of recruitment referred interested individuals to a University of Iowa website hosting a RedCap pre-screening interview [50]. The pre-screening instrument, which was conducted anonymously, is an algorithm containing 50 questions that attempted to determine whether the potential subject was over the age of 18, capable of giving informed consent in English and fit into one of four nicotine product use categories. “Controls” were defined as individuals who denied smoking more than 100 cigarettes or joints in their lifetime, denied the use of cannabis products in the past year, denied a history of substance use problems, denied currently use nicotine-containing products, and consume less than 2 alcoholic drinks per day. “Cigarette Smokers” were defined as individuals who have at least 5 pack years of consumption, currently smoke 2 or more cigarettes per day, but do not currently use ENDS. ENDS users were defined as those who use ENDS or “vape” daily, have “vaped” at least once per week for the past year, deny smoking more than 100 cigarettes in their lifetime, no use of cannabis for at least one year, no other use of tobacco-containing products. “Smokeless users” were defined as individuals who currently use chewing tobacco on a daily basis, denied smoking more than 100 cigarettes in their lifetime, no use of cannabis for at least one year, no other use of tobacco-containing products, and no ENDS use. Individuals fitting the inclusion and exclusion criteria for one of these four groups were then given an opportunity to schedule themselves for a more comprehensive in-person interview. Subjects who did not qualify for the main study were notified of their ineligibility at the completion of the screening interview.

At the time of the in-person intake interview, each potential recruit was given a written summary of the project and given a chance to ask questions. If still willing to participate, full written consent was obtained, and the study procedures were initiated. The clinical interview consisted of a 320 item interview administered through a RedCap interface comprised of questions from the PhenX Toolkit [51] that detailed demographic and substance use histories of each individual. Self-reported cigarette consumption was assessed the following question; *“How frequently have you smoked cigarettes during the past (day/week etc.)?”* Similarly, ENDS use was assessed by asking “*How frequently have you used e-cigarettes during the past (day/week, etc.)?”* Finally, smokeless tobacco consumption was assessed by asking *“How frequently have you used a smokeless tobacco product during the past (day/week, etc.)?”* For each of these questions, the participants selected one of seven potential consumption levels (e.g., “two to five cigarettes per day).

The history of alcohol consumption was similarly interrogated by a series of questions that inquired how many times they had drunk in the past month or year, their average number of drinkers per week over the past year, and who many times they drank 5 or more drinks (binge drinking) over the past two weeks. A complete copy of the interview is available upon request.

Although the interview itself was computer-administered, a research assistant remained present to answer any questions. After the interview was complete, the subject then provided urine and then was phlebotomized.

Laboratory Measures: The blood was processed into DNA and serum via our usual procedures, then stored at −20 °C and −80 °C, respectively. Enzyme-linked immunoassay (ELISA) assessments of serum cotinine and tetrahydrocannabinol (THC) levels were conducted using kits from AbNova (Taiwan) using our standard procedures [52,53]. Based on the results of these tests the data from 14 self-reported non-smoking control subjects were excluded for having serum cotinine levels >2 ng/mL. Eight self-reported daily cigarette smokers were excluded because their serum cotinine levels were <2 ng/mL. Data from six other subjects who enrolled in the study were excluded for not providing smoking use history in their intake interview (*n* = 2) or for significant discrepancies in the nicotine product use histories reported in the screening instrument as compared to the intake interviews (*n* = 6).

Secondary to the discontinuation of production of the ELISA kits during the course of this study, only 59 of the serum samples from the control subjects were examined for THC positivity. However, sera from all subjects from the smoking, ENDS, and smokeless groups were tested for THC positivity.

The determination of DNA methylation at cg05575921 and the four loci comprising the Alcohol T Score (cg02583484, cg04987734, cg09935388, and cg04583842) was conducted using universal droplet digital PCR reagents and equipment from Bio-Rad (Hercules, CA, USA) and a proprietary primer-probe set from Behavioral Diagnostics (Coralville, IA, USA) according to our previously described protocols [53,54]. The resulting DNA methylation levels are reported as percent methylation determined by the observed allele counts to a Poisson distribution. Samples failing any of these assays were removed from further analysis (*n* = 11).

Data Analysis: Statistical analyses were conducted with R version 1.3.959 and Python version 3.8.10. Comparisons between groups with respect to continuous variables were conducted using Welch’s T-Tests and ANOVA, and chi-square with respect to categorical variables.

The relationship between the Alcohol T Score (ATS) and nicotine biomarkers was analyzed using linear regression controlling for age [55,56]. The performance of each model was evaluated using adjusted *R*^2^, *p*-value, and predictor coefficients.

Heavy Alcohol Consumption (HAC) status was determined for each nicotine user by binning the ATS value at the time of the interview. The binning criteria were adapted from the average ATS value of subjects who self-reported a drinking frequency of at least 14 drinks per week within the past year (i.e., those with an ATS < 2.35 were binned as a control, and those with ATS > 2.35 were binned as a case). Subjects with missing cotinine or cg05575921 values were removed from further analyses. Target classes were numerically coded for model-building steps and evaluation.

Training and testing datasets were prepared to develop and evaluate the classifier for HAC status. The case and control subjects (*n* = 66 and *n* = 91, respectively) were split into training (70%) and testing (30%) datasets. These datasets were stratified by target class, retaining the ratio of the case:control as the original dataset. Model development and training were performed in the training set, and the final model was tested and evaluated using the test set. Using a repeated stratified cross-validation approach (10 splits and 3 repeats), a Balanced Random Forest (BRF) Classifier was built to evaluate the objective nicotine biomarkers and age for predicting HAC status in those who consume nicotine. BRF addresses the target imbalance by performing random under-sampling of the majority class in each bootstrap sample to balance the target class distribution in each itineration [57,58]. Model performance was evaluated using AUC, accuracy, sensitivity, and specificity.

## Figures and Tables

**Table 1:** Clinical and Demographic Characteristics of Nicotine Users and Control Substances.

	Smokers	ENDS	Smokeless	Control
	*n* = 108	*n* = 35	*n* = 19	*n* = 262
Age	40.9 ± 13.0	22.7 ± 4.8	36.1 ± 12.3	29.6 ± 11.4
Gender				
Male	42 (39)	13 (37)	18 (95)	79 (30)
Female	66 (61)	22 (63)	1 (5)	182 (69)
Other	-	-	-	1 (<1)
Ethnicity				
White	89 (82)	30 (86)	18 (95)	226 (86)
African American	9 (8)	2 (6)	-	6 (2)
American Indian	1 (1)	-	-	2 (<1)
Asian	3 (3)	1 (3)	-	14 (5)
Bi-racial	5 (5)	2 (6)	1 (5)	7 (3)
Other	1 (1)	-	-	7 (3)
Substance Dependence				
Nicotine	90 (83)	20 (60)	12 (63)	-
Alcohol	15 (14)	3 (9)	3 (16)	-
Both	15 (14)	2 (6)	3 (16)	-
Drinks per Week				
None	24 (22)	6 (17)	2 (11)	81 (31)
1 to 7	53 (49)	16 (46)	7 (37)	169 (65)
8 to 14	15 (14)	6 (17)	6 (32)	7 (3)
>14	12 (11)	5 (14)	4 (21)	3 (1)
cg05575921	54.5 ± 19.1	84.1 ± 5.1	84.3 ± 4.6	86.8 ± 2.8
Cotinine ng/mL	93.6 ± 32.4	78.4 ± 45.2	108.1 ± 39	-
ATS	3.7 ± 3.6	−0.5 ± 2.0	0.3 ± 2.8	−0.9 ± 2.1
ATS > 5	45 (42)	-	2 (11)	3 (1)

Mean ± Standard Deviation for Continuous Variables. *n* (%) for Categorical Variables.

## Data Availability

All data used in this study are available from the corresponding author on reasonable request.

## Supplementary Materials

The following are available online at https://www.mdpi.com/article/10.3390/epigenomes5030018/s1, Figure S1: ATS stratified by nicotine use status and substance use dependence, Figure S2: ATS distribution as a function of self-reported alcohol use, Table S1: Results for ATS multiple linear regression models, Table S2: Clinical and demographic characteristics of HAC case and control subjects.
