# Supplementary material for: Epigenetic Analyses of Alcohol Consumption in Combustible and Non-Combustible Nicotine Product Users
Source: Epigenomes. 2021 Sep 1;5(3):18. doi: 10.3390/epigenomes5030018 (PMC8594674; doi:10.3390/epigenomes5030018)

Supplementary Table S1A. Results for ATS Multiple Linear Regression Models, All Nicotine Consuming Subjects.

	Age		Age + Cotinine		Age + cg05575921	
**Predictors**	**b**	**pval**	**b**	**pval**	**b**	**pval**
Age	0.174	< 2e-16	0.17	< 2e-16	0.096	1.13e-08
Cotinine			0.005	0.427		
cg05575921					-0.092	1.06e-15
**R^2**	0.4125		0.4112		0.6105	
**pval**	< 2.2e-16		< 2.2e-16		< 2.2e-16	

Supplementary Table S1B. Results for ATS Multiple Linear Regression Models, Cigarette Users.

	Age		Age + Cotinine		Age + cg05575921	
**Predictors**	**b**	**pval**	**b**	**pval**	**b**	**pval**
Age	0.157	5.5e-11	0.157	8.67e-10	0.1	1.55e-06
Cotinine			0.004	0.70568		
cg05575921					-0.095	2.06e-10
**R^2**	0.3284		0.3229		0.5374	
**pval**	5.5e-11		4.78e-10		< 2.2e-16	

Supplementary Table S1C. Results for ATS Multiple Linear Regression Models, Smokeless Users.

	Age		Age + Cotinine		Age + cg05575921	
**Predictors**	**b**	**pval**	**b**	**pval**	**b**	**pval**
Age	0.112	3.11e-02	0.084	2.48e-02	0.117	0.0327
Cotinine			0.046	4.77e-04		
cg05575921					0.06	0.6607
**R^2**	0.2008		0.6128		0.1614	
**pval**	3.11e-02		1.97e-04		0.09535	

Supplementary Table S1D. Results for ATS Multiple Linear Regression Models, ENDS Users.

	Age		Age + Cotinine		Age + cg05575921	
**Predictors**	**b**	**pval**	**b**	**pval**	**b**	**pval**
Age	0.072	0.3190	0.072	0.33	0.022	0.8002
Cotinine			0.0003	0.97		
cg05575921					-0.151	0.0353
**R^2**	0.0007		0.03015		0.1203	
**pval**	0.3188		0.6127		0.0554	

Supplemental Table S2. Clinical and Demographic Characteristics of HAC Case and Control Subjects

	**Case**<br>N = 66	**Controls**<br>N = 91
Age	46.32 ± 11.92	29.2 ± 10.04
Gender		
Male	26 (39)	45 (49)
Female	40 (61)	46 (51)
Nicotine User Group		
Cigarette	61 (91)	44 (48)
ENDS	2 (3)	31 (34)
Smokeless	4 (6)	16 (18)
Drinks per Week		
None	13 (33)	18 (20)
1 to 7	32 (48)	41 (45)
8 to 14	8 (12)	19 (21)
> 14	10 (15)	10 (11)
Substance Dependence		
Nicotine	58 (88)	61 (67)
Alcohol	12 (18)	8 (9)
Both	12 (18)	7 (8)
cg05575921	47.87 ± 17.77	76.30 ± 14.28
Cotinine ng/ml	102.08 ± 30.32	85.74 ± 39.65
ATS	6.05 ± 2.23	- 0.31 ± 1.59

Mean ± Standard Deviation for Continuous Variables

N (%) for Categorical Variables

Supplemental Figure S1. ATS Stratified by Nicotine Use Status and Substance Use Dependence.

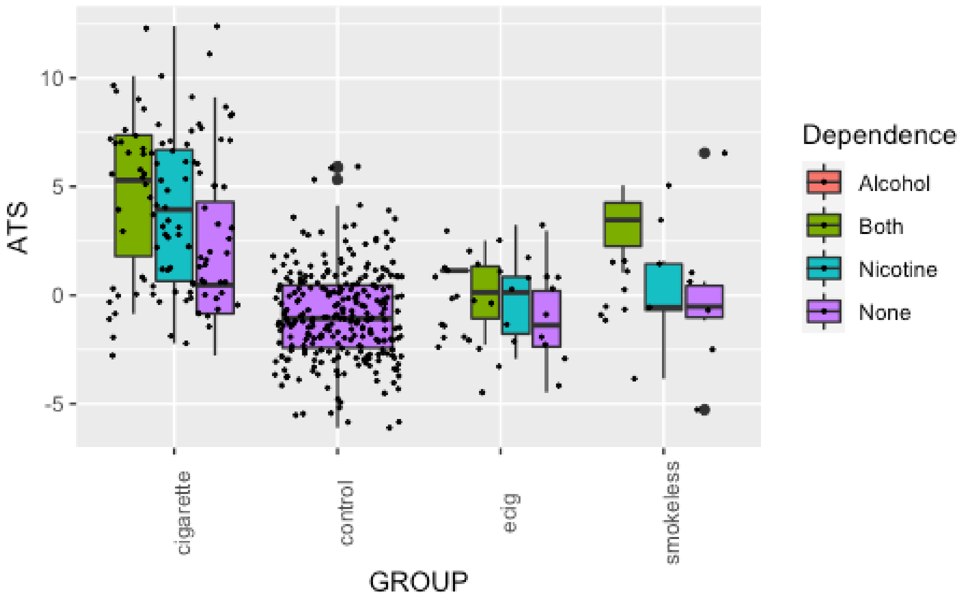

Supplementary Figure S2. ATS distribution as a function of Self-reported Alcohol Use.

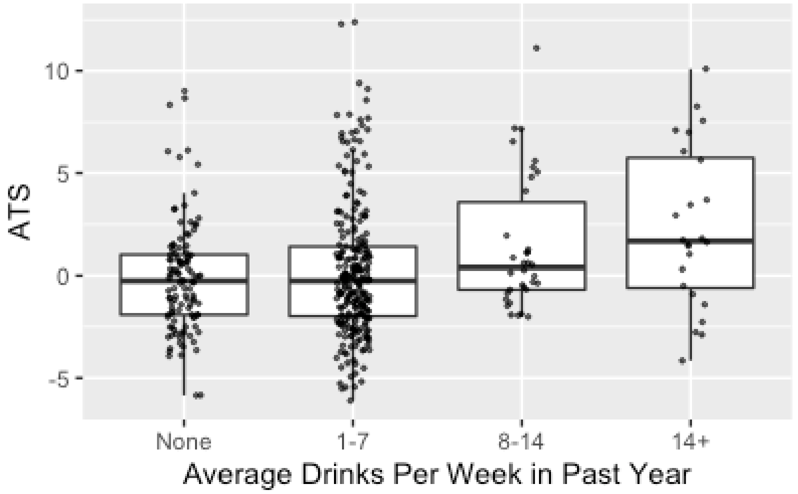

Supplement: Supplementary file 1 [file epigenomes-05-00018-s001.zip › epigenomes-1337216-supplementary.pdf]
